# Dasatinib and Prednisolone Induction Therapy for a Case of Philadelphia Chromosome-Positive Acute Lymphoblastic Leukemia with Dilated Cardiomyopathy Accompanied by Life-Threatening Ventricular Tachycardia

**DOI:** 10.1155/2017/4027908

**Published:** 2017-02-23

**Authors:** Mitsutaka Nishimoto, Hirohisa Nakamae, Kana Matsumoto, Kunihiko Morita, Yuki Koga, Dai Momose, Masayuki Hino

**Affiliations:** ^1^Hematology, Graduate School of Medicine, Osaka City University, 1-4-3 Asahi-machi, Abeno, Osaka, Japan; ^2^Department of Clinical Pharmaceutics, Faculty of Pharmaceutical Sciences, Doshisha Women's College of Liberal Arts, Kodo, Kyotanabe, Kyoto, Japan; ^3^Department of Cardiovascular Medicine, Graduate School of Medicine, Osaka City University, 1-4-3 Asahi-machi, Abeno, Osaka, Japan; ^4^Department of Hematology, Osaka General Hospital, West Japan Railway Company, 1-2-22 Matsuzaki-cho, Abeno, Osaka, Japan

## Abstract

A 56-year-old man being treated for dilated cardiomyopathy presented with epigastralgia. He was diagnosed with ventricular tachycardia and Philadelphia chromosome-positive acute lymphoblastic leukemia. After treating incessant ventricular tachycardia, we commenced induction therapy for leukemia with dasatinib and prednisolone to minimize toxicity towards cardiomyocytes and the cardiac conduction system. Although dasatinib was temporarily withheld because of a recurrence of ventricular tachycardia, we rechallenged dasatinib while using bisoprolol and amiodarone and achieved a complete hematological response three weeks later. Although drug interactions between dasatinib and amiodarone were of concern, the blood concentration of each drug remained within the safe range after concomitant use, and there were no adverse cardiac effects such as QT prolongation after rechallenging dasatinib. Induction therapy with dasatinib and prednisolone may be an acceptable therapeutic option for Philadelphia chromosome-positive acute lymphoblastic leukemia with severe cardiac complications.

## 1. Introduction

Dasatinib, a second-generation tyrosine kinase inhibitor (TKI), has profoundly improved the prognosis for Philadelphia chromosome-positive leukemia [1]. Combined with steroids, dasatinib has been used to effectively treat Philadelphia chromosome-positive acute lymphoblastic leukemia (Ph^+^ ALL) [2]. It is well known that TKIs have cardiovascular toxicity and can cause ventricular tachycardia (VT) because of QT prolongation [3–5]. In a randomized phase-3 trial, drug-related adverse cardiac effects occurred in 17 of 258 leukemia patients treated with dasatinib [6]. Dasatinib was also discontinued after QT prolongation in one patient among 26 Japanese leukemia patients [7]. In patients with advanced solid tumors, a high dasatinib blood concentration has been associated with prolongation of the QTc interval [8]. We should be aware of adverse cardiac effects when using TKIs. However, there are few data regarding treatments for Ph^+^ ALL patients with cardiomyopathy or life-threatening ventricular arrhythmias. Although it has been reported that drug interactions between dasatinib and amiodarone can occur [9], there has not yet been much clinical data. Herein, we describe induction therapy with dasatinib plus prednisolone with concomitant use of bisoprolol and amiodarone for a patient with Ph^+^ ALL who had dilated cardiomyopathy and incessant VT.

## 2. Case

**Figure 1:**
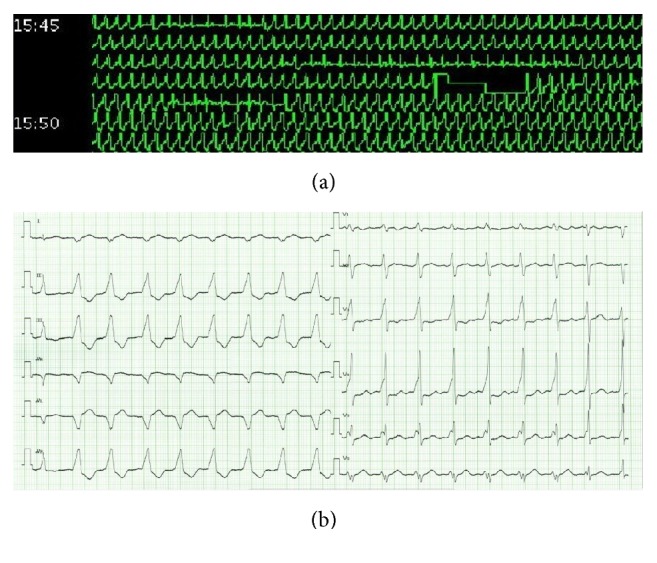
The 24-hour and twelve-lead electrocardiograms on admission. The 24-hour electrocardiogram shows a ventricular arrhythmia persisting for more than five minutes, and the twelve-lead electrocardiogram on admission shows incessant ventricular tachycardia.

**Figure 2:**
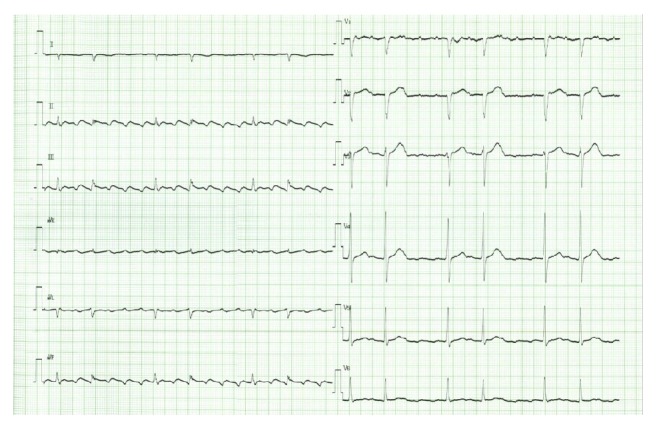
Electrocardiogram on the 22nd day after dasatinib retreatment. Potentially fatal ventricular arrhythmias have been replaced by atrial flutter.

**Figure 3:**
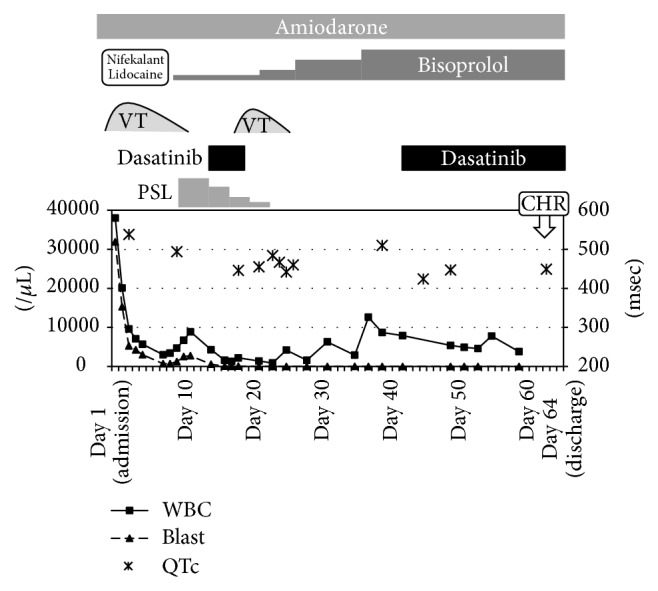
Clinical course before and after dasatinib treatment. VT, ventricular tachycardia; PSL, prednisolone; CHR, complete hematological response.

A 56-year-old man who had been treated for dilated cardiomyopathy and atrial fibrillation presented at the hospital with epigastralgia. His electrocardiogram showed nonsustained VT, which developed into VT storms even after starting amiodarone (Figure 1). Nifekalant hydrochloride and lidocaine were also administered, and the ventricular arrhythmias then decreased, and atrial flutter was seen. After the hemodynamics had been stabilized, we started bisoprolol with a dose of 0.625 mg daily. While treating the cardiac complications, we assessed the patient's blood sample. Severe anemia and blastoid cells were observed in the peripheral blood. The bone marrow contained 96% lymphoid blast cells with a 46, XY, t(9;22)(q34;q11.2) karyotype, and minor bcr-abl mRNA transcripts were detected at 7.0 × 10^5^ copies/*μ*g RNA. The patient was diagnosed with Ph^+^ ALL and received induction therapy with dasatinib 50 mg twice daily and prednisolone 100 mg daily to minimize toxicity towards cardiomyocytes and the cardiac conduction system. We started dasatinib at a lower dose because of concerns about cardiotoxicity. Five days after initiating dasatinib and prednisolone, dasatinib was discontinued because of increments in nonsustained VT although the QTc interval had not become significantly prolonged (455 ms). We gradually increased the dose of bisoprolol to 3.75 mg daily. After being withdrawn for 22 days, dasatinib was rechallenged, and we could continue without the appearance of nonsustained VT or the prolongation of QTc intervals (Figures 2 and 3). The serum concentration of dasatinib one hour after oral administration (*C*_1 hr_) and the plasma concentrations of amiodarone and desethylamiodarone approximately 12 hours after oral administration on the 10th day of concomitant use were 45.7 ng/mL, 660.6 ng/mL, and 449.0 ng/mL, respectively. Three weeks after retreatment with dasatinib, we confirmed a complete hematological response and a 2-log reduction in minor bcr-abl mRNA transcripts in the patient's bone marrow (Table 1). He wished to be discharged from hospital and refused a dose-escalation of dasatinib or chemotherapy. On the 88th day after his admission, blastoid cells appeared in the peripheral blood. We discontinued dasatinib at that time because of the refractoriness of his leukemia. Although VT recurred and lasted for five days, it disappeared following the initiation of steroid pulse therapy and furosemide. After that, VT did not recur. We did not add any other TKIs or chemotherapy except for dexamethasone and the short-term use of mercaptopurine. Eventually, he died from leukemia on the 183rd day after his admission.

## 3. Discussion

We encountered a Ph^+^ ALL patient with dilated cardiomyopathy accompanied by incessant VT and achieved a complete hematological response with dasatinib and prednisolone treatment without worsening the cardiomyopathy and ventricular arrhythmias.

Since chemotherapy including anthracycline confers a high risk of congestive heart failure [10], we avoided cardiotoxic antitumor agents and used dasatinib plus prednisolone instead. Dasatinib may also induce congestive heart failure because of fluid retention, pulmonary arterial hypertension, and ventricular arrhythmias [11]. In particular, we should be aware of adverse cardiac effects in a patient who has cardiac complications prior to therapy. In our case, we achieved a complete hematological response without fluid retention, pulmonary arterial hypertension, or ventricular arrhythmias, although this might have been affected by the short duration of dasatinib therapy.

We also continued dasatinib without exacerbating the VT. Spechbach et al. described a case of ventricular arrhythmia induced by dasatinib and the successful continuation of dasatinib with concomitant use of antiarrhythmic agents [12]. In the current case, we used bisoprolol and amiodarone to control VT during dasatinib therapy. Bisoprolol restrains excessive myocyte activity by inhibiting the myocardial beta 1 receptor [13], and amiodarone prolongs phase 3 cardiac action potential [14]. Dasatinib is not thought to directly harm cardiac mitochondrial function but to affect Purkinje-fiber assays and human ether-a-go-go-related gene potassium ion channels (HERG K^+^) [5, 15], which is why we might have been able to continue dasatinib without increments in VT during treatment with bisoprolol and amiodarone.

Dasatinib and amiodarone are both thought to be metabolized mainly by CYP3A4, and the concurrent use of these two drugs can elevate the concentration of each drug [9]. The actual dasatinib *C*_1 hr_ was not particularly high, and the plasma concentration of amiodarone stayed within the safe range. It was speculated that the drug concentrations might have not reached a high enough range to inhibit CYP3A4 [16, 17].

Since a low plasma concentration of dasatinib can cause the bcr-abl T315I point mutation, we should use a substantial dose of dasatinib, if possible [18]. In the present case, there was a possibility that T315I mutations had occurred at the time of relapse, though this could not be assessed. The pharmacokinetics of dasatinib vary widely in individuals [8, 18]. Thus, we could consider a dose-escalation of dasatinib to prevent the occurrence of bcr-abl point mutations while monitoring the dasatinib blood concentration. We should have increased the dose of dasatinib to 140 or 180 mg daily and tried to maintain the dasatinib Cmax above 50 ng/mL [18, 19].

In conclusion, induction therapy with dasatinib and prednisolone may be an acceptable therapeutic option for Ph^+^ ALL patients with dilated cardiomyopathy and life-threatening ventricular arrhythmias. We can consider escalating the dose of dasatinib while monitoring its blood concentration. More cases should be accumulated to establish the optimal therapy for Ph^+^ ALL with severe cardiac complications.

## Figures and Tables

**Table 1:** Laboratory data.

	Admission to hospital	Before retreatment with dasatinib	22 days after retreatment
Peripheral blood samples			
Leukocytes (×10^2^/*μ*L)	380	79	78
Neutrophils (%)	4	67	65
Lymphocytes (%)	7	28	15
Monocytes (%)	0	2	7
Blastoid cells (%)	84	0	0
Hemoglobin (g/dL)	7.6	9.4	8.2
Platelet (×10^4^/*μ*L)	5.4	19.8	6.8
Bone marrow samples			
Total nucleated cells (/*μ*L)	74500		11000
Blastoid cells (%)	96		0.4
Minor bcr-abl mRNA (copy/*μ*g RNA)	7.0 × 10^5^		7.9 × 10^3^
Karyotype by G-banding	46XY, t(9;22)(q34;q11.2) [17/20]		46XY [20/20]
